# A Small Dose With Large Movements: Suspected Gabapentin-Associated Mixed Hyperkinetic Movement Disorder in End-Stage Renal Disease: A Case Report

**DOI:** 10.7759/cureus.116598

**Published:** 2026-09-20

**Authors:** Kian Memari, Daniela Prado Escobar, Sergio A Rodriguez, Eva Gallo, Joren Manuel, Peter Cohen, Shane Williams, Lissette P Lazo

**Affiliations:** 1 Family Medicine Residency, Palmetto General Hospital, Hialeah, USA; 2 Medicine, Nova Southeastern University Dr. Kiran C. Patel College of Osteopathic Medicine, Clearwater, USA; 3 Family Medicine, Nova Southeastern University Dr. Kiran C. Patel College of Osteopathic Medicine, Fort Lauderdale, USA

**Keywords:** chorea, choreoathetosis, diabetic neuropathy, drug-induced movement disorder, end-stage renal disease, gabapentin, neurotoxicity, renal dosing

## Abstract

Gabapentin is eliminated almost entirely by the kidneys, and markedly reduced clearance in end-stage renal disease (ESRD) can predispose patients to neurotoxicity. Myoclonus is the most frequently reported gabapentin-associated movement disorder, whereas choreiform and other dyskinetic phenotypes are less common.

We present a 77-year-old Hispanic man with ESRD, hypertension, diabetes mellitus with neuropathic pain, and macrocytic anemia who presented to the emergency department for generalized weakness, not for an abnormal movement complaint. Collateral history subsequently established approximately two months of gabapentin exposure at 100 mg by mouth twice daily and approximately one month of new involuntary movements. Examination after transient lorazepam-related somnolence revealed repeated bilateral upper-extremity and truncal jerks with a slower back-and-forth truncal sway, preserved cranial nerve function, 5/5 strength, and no focal neurologic deficit. Because the contemporaneous description repeatedly used the terms "jerking" and "rhythmic," the available phenomenology was more compatible with prominent myoclonic activity than with definitive chorea, which is characteristically irregular and non-rhythmic; a mixed hyperkinetic movement disorder is therefore the most defensible descriptive classification. Gabapentin was discontinued, and the abnormal movements and altered affect resolved within approximately 48 hours. The available source record confirms ESRD and a right upper-extremity arteriovenous (AV) fistula but does not preserve the maintenance hemodialysis schedule, timing of the last treatment, whether hemodialysis occurred during recovery, serum gabapentin concentration, or the complete contemporaneous metabolic panel. Accordingly, the clinical course supports suspected gabapentin-associated neurotoxicity but does not establish causality or exclude dialysis-related drug removal or another metabolic contributor.

This case emphasizes careful medication reconciliation, renal- and dialysis-specific dosing, descriptive movement phenomenology, and cautious causal attribution in patients with kidney failure who develop new hyperkinetic movements.

## Introduction

Gabapentin is a structural analog of gamma-aminobutyric acid that is widely used for neuropathic pain, epilepsy, postherpetic neuralgia, and other pain syndromes. It does not directly activate gamma-aminobutyric acid receptors; instead, it binds the alpha-2-delta subunit of voltage-gated calcium channels and decreases excitatory neurotransmitter release, including glutamate and substance P [1,2].

Neurologic adverse effects of gabapentin include dizziness, somnolence, fatigue, ataxia, diplopia, tremor, and dysarthria. Hyperkinetic movement disorders are uncommon but increasingly recognized. A 2023 literature review identified 204 individuals with reported gabapentin-associated movement disorders; myoclonus was the dominant phenotype, while dyskinesia and other abnormal movements were less frequent [3]. Importantly, reported doses ranged from 100 to 9,600 mg/day, indicating that absolute dose alone does not determine risk [3].

Renal function is central to gabapentin safety because the drug is not substantially metabolized and is excreted unchanged by the kidneys. In advanced chronic kidney disease and end-stage renal disease (ESRD), clearance is markedly reduced and dosing must be individualized to renal function and dialysis exposure [4]. In anuric patients, gabapentin has a very prolonged elimination half-life between hemodialysis treatments, while hemodialysis substantially increases clearance [5]. Therefore, the timing of dialysis can influence both toxicity and recovery.

Gabapentin neurotoxicity in renal failure is particularly associated with encephalopathy and myoclonus, including cases that improve after drug discontinuation and dialysis [6]. Choreiform reactions have also been described [7-9]. Because chorea is irregular and unpredictable whereas myoclonus consists of brief shock-like jerks, precise phenomenologic description is essential when reviewing a suspected medication-associated movement disorder. We report a low absolute-dose gabapentin-associated mixed hyperkinetic syndrome in an older patient with ESRD, emphasizing both the diagnostic signal and the limitations imposed by incomplete dialysis and metabolic data.

## Case presentation

A 77-year-old Hispanic man with hypertension, diabetes mellitus complicated by neuropathic pain, ESRD with a right upper-extremity arteriovenous (AV) fistula, and macrocytic anemia presented to the emergency department with generalized weakness. The weakness was described as constant and exacerbated by movement and exertion. Abnormal involuntary movements were not the presenting complaint. On arrival, the patient was agitated and received lorazepam, which produced transient somnolence and initially limited observation of the abnormal movements.

Collateral history from his wife later clarified that he had longstanding baseline restlessness but had developed a distinctly new involuntary movement syndrome during the preceding month. She also reported that he had been taking gabapentin 100 mg by mouth twice daily for approximately two months for diabetic neuropathic pain. Thus, gabapentin exposure preceded the new movements by approximately one month.

The available record confirms ESRD and a functioning right upper-extremity AV fistula; however, the dialysis flow sheet and contemporaneous nephrology documentation were not available in the source materials reviewed for this report. Consequently, whether the patient was actively receiving maintenance hemodialysis, his usual dialysis schedule, the timing of the last dialysis treatment relative to symptom onset and admission, and whether hemodialysis occurred during the approximately 48-hour recovery interval could not be verified. These details were not inferred.

On admission, blood pressure was 100/58 mmHg and respiratory rate was 18 breaths/min. Cardiovascular examination documented a regularly irregular rhythm at a normal rate without murmurs, rubs, or gallops. The AV fistula had a palpable thrill. On pulmonary examination, bilateral breath sounds were present without wheezes, rhonchi, or crackles; no focal reduction in air entry was documented. The abdomen was soft, non-tender, and non-distended.

Neurologically, the patient was disoriented to person, place, and time during the initial evaluation but could follow commands. Cranial nerves were intact. Strength was 5/5 bilaterally in the upper and lower extremities, and no focal neurologic deficit was identified. After the sedative effect of lorazepam diminished sufficiently for observation, the abnormal movements were described as continuous over the bedside observation period and bilateral, with repeated brief jerks of both upper extremities and the trunk together with a slower back-and-forth truncal sway (Table 1). The original bedside descriptions used the words "jerking" and "rhythmic." There was no documentation of a flowing, random, non-stereotyped migration of movements between body regions. On phenomenologic review, the repeated brief jerks favored a myoclonic component, while the slower truncal movement suggested an additional dyskinetic component. Because no video recording, surface electromyography-electroencephalography polygraphy, or movement-disorders specialist description was available, the movements could not be classified confidently as pure chorea or choreoathetosis. Therefore, we used the descriptive term mixed hyperkinetic movement disorder with prominent myoclonic features.

**Table 1:** Pertinent clinical and diagnostic findings AV: Arteriovenous; CBC: Complete blood count; ESRD: End-stage renal disease

Clinical domain	Observed finding	Interpretation/Limitation
Presenting complaint	Generalized weakness, constant and worse with movement/exertion	Abnormal movement was identified during evaluation and collateral history; it was not the chief complaint.
Gabapentin exposure	100 mg by mouth twice daily for approximately two months	New abnormal movements began approximately one month after gabapentin initiation.
Renal/dialysis status	ESRD; right upper-extremity AV fistula with palpable thrill	Maintenance hemodialysis status/schedule, last treatment timing, and dialysis during recovery were not recoverable from the reviewed source materials.
Mental status	Disoriented to person, place, and time; followed commands	Lorazepam-related somnolence transiently limited the initial movement examination.
Motor/cranial nerves	5/5 strength in all extremities; cranial nerves intact; no focal deficit	Argues against a focal motor syndrome but does not by itself exclude structural or metabolic disease.
Movement phenomenology	Repeated brief bilateral upper-extremity and truncal jerks plus slower back-and-forth truncal sway; bedside record used "rhythmic"	Prominent myoclonic features; insufficient documentation to defend definitive chorea/choreoathetosis.
Available laboratory history	Macrocytic anemia; cobalamin and folate normal; prior bone marrow biopsy reportedly negative	Contemporaneous CBC and metabolic/electrolyte values were not recoverable.
Management/outcome	Gabapentin discontinued; movements and altered affect resolved within approximately 48 hours	Positive dechallenge supports suspected drug association, but dialysis-related clearance or correction of another metabolic factor cannot be excluded.

The diagnostic data recoverable from the source record were limited. Macrocytic anemia was documented, with normal cobalamin and folate levels; a prior bone marrow biopsy performed during evaluation for possible myelodysplastic syndrome was reportedly negative. The preserved cranial nerve examination, symmetric full strength, and absence of focal deficits reduced concern for a large focal motor lesion but did not exclude structural disease. The reviewed source materials did not preserve contemporaneous values for the complete blood count (CBC) indices, sodium, potassium, bicarbonate, glucose, blood urea nitrogen, creatinine, calcium, magnesium, phosphorus, liver enzymes, thyroid-stimulating hormone, or serum gabapentin concentration. They also did not document brain imaging or electroencephalography in the materials available to the authors. Accordingly, the manuscript does not claim that structural, metabolic, or epileptic causes were definitively excluded.

Given the temporal relationship between gabapentin exposure and the new movement syndrome, the high-risk renal physiology, and the known association between gabapentin and myoclonus in renal failure, gabapentin-associated neurotoxicity was suspected and gabapentin was discontinued. Rechallenge was not performed. Within approximately two days, the abnormal movements resolved and the patient's affect and energy returned to baseline; he was discharged without focal neurologic deficits. Because dialysis timing during this interval is unavailable, the improvement represents a supportive dechallenge response but cannot be attributed solely to gabapentin discontinuation.

## Discussion

This case is best interpreted as a suspected gabapentin-associated mixed hyperkinetic movement disorder in a patient with ESRD rather than as definitively proven choreoathetosis. The original clinical record repeatedly used the terms "jerking" and "rhythmic," which are not typical defining descriptors of chorea. Chorea is irregular, unpredictable, and non-rhythmic, while myoclonus is characterized by brief shock-like jerks. In the 2023 review of gabapentin-associated movement disorders, myoclonus accounted for most reported cases and had the strongest evidence of association [3]. The observed repeated brief jerks, therefore, made myoclonus the leading phenomenologic component in this patient; the slower truncal sway suggested a mixed rather than purely myoclonic syndrome.

The renal context materially increases biologic plausibility. Gabapentin is cleared by renal excretion, and accumulation can occur when kidney function is severely impaired [4]. Pharmacokinetic study in anuric subjects demonstrated an average gabapentin elimination half-life of approximately 132 hours on non-hemodialysis days, compared with approximately four hours during hemodialysis; roughly 35% of an administered dose was recovered in dialysate [5]. These data explain why dialysis timing is crucial when interpreting both toxicity and rapid recovery. A dose of 200 mg/day is low by usual adult outpatient standards but may still produce clinically important exposure in ESRD if it is not matched to renal clearance and dialysis scheduling. The broader literature also includes gabapentin-associated movement disorders at doses as low as 100 mg/day [3], so the teaching point is impaired clearance rather than an unprecedented absolute dose.

Alternative causes deserve explicit consideration. Uremia and electrolyte abnormalities can produce encephalopathy, tremor, asterixis, and myoclonus; hyperglycemia can produce choreiform movements; seizures may manifest with repetitive jerks; and structural neurologic disease can cause new abnormal movements. In this patient, the bilateral movement pattern, symmetric 5/5 strength, intact cranial nerve examination, and absence of focal neurologic deficits made a large focal lesion less clinically apparent. The subacute onset after gabapentin exposure and complete resolution after withdrawal further supported a medication-associated syndrome. However, because the contemporaneous metabolic values, dialysis timing, serum gabapentin concentration, neuroimaging, and electroencephalography were not recoverable, these alternatives cannot be considered definitively excluded.

The clinical course therefore supports association rather than proof of causation. Improvement within approximately 48 hours after gabapentin discontinuation is a positive dechallenge signal, but gabapentin is dialyzable [5,6]. If hemodialysis occurred during the recovery period, dialysis could have materially accelerated drug elimination and contributed to symptom resolution. Similarly, correction of an undocumented metabolic abnormality could have contributed. For these reasons, the revised manuscript uses "suspected" throughout and avoids attributing recovery solely to medication discontinuation.

From a practical standpoint, an older patient with ESRD who develops a new hyperkinetic movement should prompt three parallel actions: a precise descriptive movement examination, immediate medication reconciliation with attention to renally eliminated neuroactive drugs, and targeted evaluation for metabolic and structural mimics. For gabapentin specifically, the prescribed dose should be interpreted together with kidney function and dialysis schedule rather than in isolation. When toxicity is suspected, gabapentin should be held while reversible competing causes are assessed; hemodialysis may be relevant when clinically indicated because it substantially increases gabapentin clearance [5,6].

This report is limited by the retrospective nature and incompleteness of the source record available for manuscript preparation. The exact maintenance hemodialysis status and schedule, timing of the last dialysis treatment before presentation, and whether dialysis occurred during the 48-hour recovery period were not recoverable. These omissions prevent separation of spontaneous renal drug clearance from dialysis-assisted clearance.

The contemporaneous CBC, electrolyte profile, glucose, uremic indices, calcium, magnesium, phosphorus, thyroid studies, serum gabapentin concentration, brain imaging, and electroencephalography were also not available in the reviewed materials. Accordingly, metabolic, structural, and epileptic mimics cannot be stated to have been formally excluded. Finally, the movement phenomenology is based on bedside documentation without video, electrophysiologic characterization, or specialist movement-disorder examination; therefore, this report intentionally uses a descriptive mixed hyperkinetic classification rather than definitive choreoathetosis.

## Conclusions

Gabapentin-associated neurotoxicity should be considered when a patient with ESRD develops new involuntary movements, even when the prescribed absolute dose appears low. In this case, the documented repeated brief bilateral jerks favor a prominent myoclonic component rather than definitive choreoathetosis, with an additional slower truncal dyskinetic movement.

The approximately 48-hour resolution after gabapentin withdrawal supports a suspected medication-associated adverse effect but does not establish causality because the dialysis schedule, dialysis exposure during recovery, and complete metabolic evaluation were not recoverable. Clinicians should pair medication review with renal- and dialysis-specific dosing assessment and evaluation for reversible metabolic and neurologic mimics before assigning causation.
